# Aerosol jet printing polymer dispersed liquid crystals on highly curved optical surfaces and edges

**DOI:** 10.1038/s41598-022-23292-9

**Published:** 2022-11-02

**Authors:** Matthew Davies, Matthew J. Hobbs, James Nohl, Benedict Davies, Cornelia Rodenburg, Jon R. Willmott

**Affiliations:** 1 Sensor Systems Group, Department of Electronic and Electrical Engineering, University of Sheffield, Sheffield, UK; 2 Department of Materials Science and Engineering, University of Sheffield, Sheffield, UK

**Keywords:** Optical materials and structures, Electronic devices

## Abstract

We demonstrate a new technique for producing Polymer Dispersed Liquid Crystal (PDLC) devices utilising aerosol jet printing (AJP). PDLCs require two substrates to act as scaffold for the Indium Tin Oxide electrodes, which restricts the device geometries. Our approach precludes the requirement for the second substrate by printing the electrode directly onto the surface of the PDLC, which is also printed. The process has the potential to be precursory to the implementation of non-contact printing techniques for a variety of liquid crystal-based devices on non-planar substrates. We report the demonstration of direct deposition of PDLC films onto non-planar optical surfaces, including a functional device printed over the 90° edge of a prism. Scanning Electron Microscopy is used to inspect surface features of the polymer electrodes and the liquid crystal domains in the host polymer. The minimum relaxation time of the PDLC was measured at 1.3 ms with an 800 Hz, 90 V, peak-to-peak (Vpp) applied AC field. Cross-polarised transmission is reduced by up to a factor of 3.9. A transparent/scattering contrast ratio of 1.4 is reported between 0 and 140 V at 100 Hz.

## Introduction

Liquid Crystal (LC) devices are ubiquitous with optical devices such as displays^1–3^, light shutters/valves^4,5^, spatial light modulators^6,7^, and tuneable filters^8,9^. The electro-optical characteristics of LCs make them ideally suited to these applications. Their utility arises from their unusual molecular order, strong response to electric fields and the macroscopic optical effects produced as a result of these properties. LCs are birefringent; light experiences a different refractive index when passing through them depending on its polarisation^10^. Recent developments in Organic Light Emitting Diode (OLED) technology has begun to displace Liquid Crystal Displays (LCDs) from their more traditional consumer applications^11^. However, LCs are still dominant in the field of light modulation and holography^12–15^.

Polymer Dispersed Liquid Crystal (PDLC) is a composite material that has electrically variable light scattering properties. Its applications are varied and include smart windows^16^, variable gratings^15,17^, gas sensing^18^ and light modulation^19–21^. Although PDLC devices can be produced with flexible substrates^22–25^, the traditional production methods and device structure restrict their geometries which limits their applications. The application space would be significantly expanded if they could be produced on a single, non-planar, substrate.

Aerosol Jet Printing (AJP) is an emergent technology in the field of additive manufacturing and 3D printing^26^. It is a fine feature production method, a characteristic which is paramount in the age of device miniaturisation and lab-on-a-chip systems. AJP affords direct deposition of any sufficiently inviscid solution-processable material and has been used to demonstrate a host of active and passive electrical components^27–31^. This allows implementation of miniaturised optoelectronic components without constraints imposed by traditional integrated circuit designs with silicon. AJP produce features with a minimum spatial resolution of 10 µm^32^. This allows for detailed patterns to be printed with high precision and with minimal processing steps, due to the direct write nature of the process. The rapid prototyping nature of the AJ printing process coupled with large freedom in substrate material and shape^33^ makes the implementation of designs for printed active components flexible, in both senses of the word, and convenient.

We use AJP to produce the first fully printed PDLC device based on conductive polymer electrodes. The full device is deposited onto a single substrate and characterised for its electro-optical properties. To our knowledge, this is the first report of a LC device that is printed using AJP. We also report functional devices printed directly onto curved and angled glass surfaces, to demonstrate the utility and potential for the AJP process in LC optical devices.

## Background

### Operational principle of PDLC

A basic function of PDLC devices is as diffusers which can be switched from a scattering to an optically clear state through the application of an electric field (or vice versa)^34^. The utility can be increased with the addition of dichroic dyes, referred to as a guest–host mixture^35,36^.

**Figure 1:**
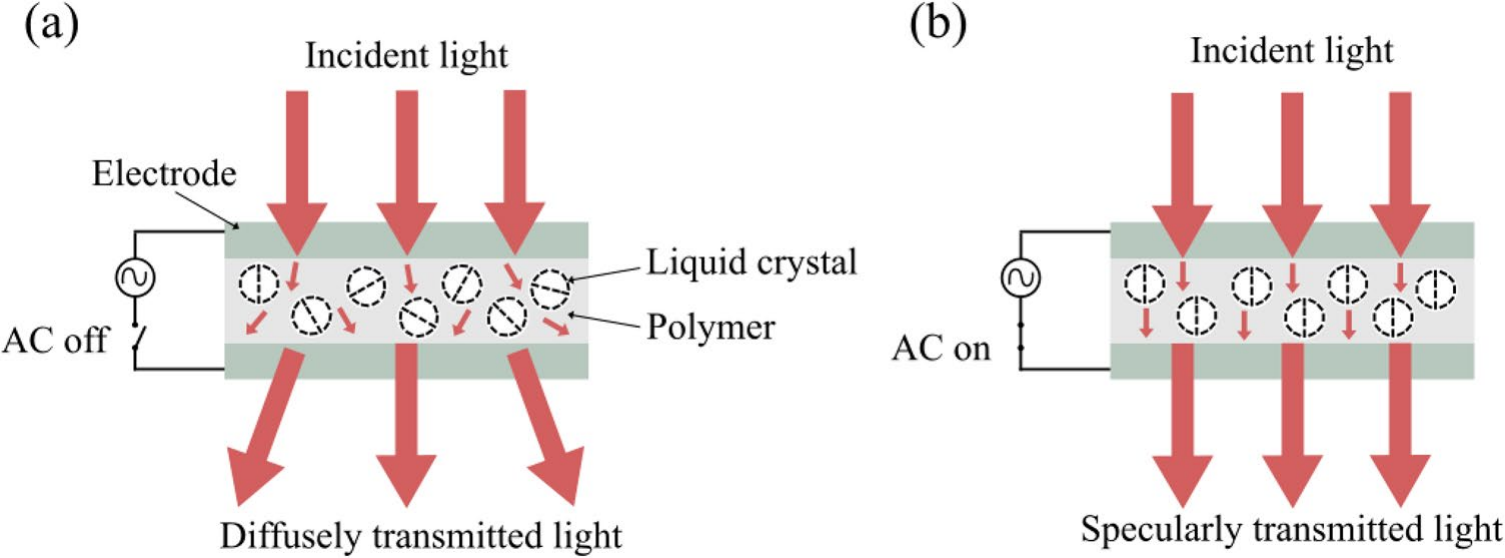
Cross-sectional schematic view of PDLC operation: (**a**) No applied AC electric field. LC droplet orientation is random and transmitted light is scattered; (**b**) AC electric field is applied, the PDLC becomes optically transparent as the LC molecules in the droplets align with the field. Transmitted light is no longer scattered.

Figure 1 shows a cross-sectional representation of a traditional PDLC cell. It operates by utilising the refractive index mismatch between the LC and the polymer matrix within which it is contained. Each micron scale droplet of LC has highly localised directional order of its constituent rod-like (nematic) molecules^37^ and the individual droplets are randomly orientated relative to each other. The result is that there is strong contrast between the LC and the polymer refractive indices which causes random scattering of light due to multiple instances of refraction at the LC-polymer interfaces. This optical phenomenon is analogous to the scattering of light due to the refractive index difference between fat droplets and water in milk. In PDLC, however, the local molecular ordering of each droplet also repolarises transmitted light. This allows a portion of scattered light to pass through pairs of crossed linear polarisers when the PDLC is placed between them.

The PDLC is sandwiched between two transparent, conductive electrodes to enable a clear state of the device to be achieved. The LC molecules align with the field when a uniform, alternating current (AC) electric field is applied to the electrodes; their refractive index matches the host polymer allowing light to pass unhindered. The LC domains inside the PDLC no longer repolarise light in the on-state because they are directed along a single axis of transmission parallel to the applied electric field.

PDLC films can be produced in several ways; typically through photopolymerisation, thermally and solvent induced phase separation (PIPS^22^, TIPS^38^ and SIPS^39^ respectively). For all these methods, phase separation (either induced by polymerisation of a monomer or through evaporation of a solvent) forces droplets of LC to form inside the polymer which adopts a spongey texture saturated with LC domains. SIPS is the technique used in this work. Both the LC and polymer are dissolved in a solvent together and they separate as the solvent evaporates^37^.

PDLC devices are traditionally produced by filling a cell with a polymer–LC pre-mixture prior to phase separation. The cell is formed of two substrates that are coated with a transparent, conductive material, typically Indium-Tin Oxide (ITO) and parted with a spacer^34^. The mixture is then treated to induce phase separation and the PDLC is attained.

Voltage dependent transmittance/opacity measurements are usually reported for PDLCs in the literature ^34^. This specifically refers to how the proportion of light transmitted along its original path, i.e. not scattered, varies with applied voltage. This is an ambiguous term when not clearly defined, especially to the reader unfamiliar with PDLC characterisation. Therefore, we explicitly define and adopt our meaning of transparency below.

### Clarification of terminology

Transmittance can imply all light transmitted (not reflected or absorbed) through a surface of a material (i.e. radiant flux), regardless of scattering (A + T + R = 1 Eq. 1). Transparency is used in this work to refer to light that is transmitted, but not scattered. The higher degree of scattering, the less transparent the material is said to be. Opacity and transmittance are appropriate terms when describing PDLCs that are doped or structured such that they exhibit variable absorption or reflection of light. The PDLCs reported in this work are not doped and, as such, are at no time referred to as being opaque. Transmission, as referred to in this text, is used herein to describe all light not absorbed or reflected and transparency as specular transmission. Specular transmission is light that passes through the device along the optical axis.

1 $$A + T + R = 1$$

Equation (1) shows the relationship between absorbed, transmitted and reflected light incident ona surface, where, *A* is absorption, *T* is transmittance and *R* is reflectance.

### Aerosol jet deposition

Our devices were printed using an Optomec Aerosol Jet 300^26^. In AJ deposition, two gas flows act to deposit material continuously in the form of micron scale droplets. One of these transports the droplets from a vial in which they are aerosolised by sonication; the other forms a coaxial sheath that focuses the stream prior to deposition through a ceramic nozzle. The device used to create the aerosol is known as an atomiser. The overall effect is the non-contact deposition of solution processable materials in a process that has very little dependence on substrate material or shape.

**Figure 2:**
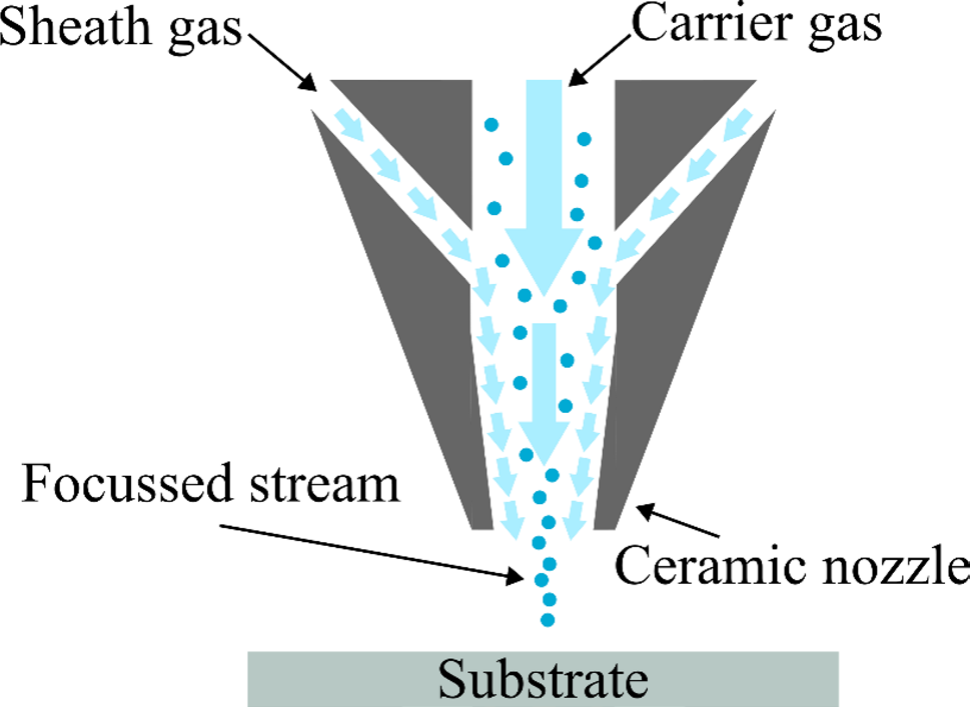
Diagrammatical representation of the aerosol jet deposition head.

The gas flows described are shown in Fig. 2. Although AJ deposition has been extensively applied to the field of flexible electronics^28,40,41^, to the best of our knowledge, AJ deposition of LC has not been investigated. AJ deposition is intimately compatible with SIPS because it is necessary to use a solvent in order to facilitate aerosolisation prior to deposition. Hence, SIPS is the method used in the work herein.

## Methods

The LC chosen for our study of AJP PDLC was 7cb (98% 4′-Heptyl-4-biphenylcarbonitrile) (Merck 24,859,917) because it is widely available with a relatively high temperature clearing point, the point at which thermal energy disrupts the molecular order and the LC is no longer birefringent (42.8 °C)^42^. This enabled room temperature operation of our devices. The host polymer was PS:PMMA: PS (Polymer Source inc P40168-SMMAS). The 7cb and PS:PMMA: PS were mixed 3:2 by weight and dissolved in ethyl acetate (> 99.5% Merck 319,902) in a 2% concentration. This ratio of LC to polymer was chosen because lower relative quantities of LC (1:1) gave poor cross-polarised transmission, suggesting a lack of LC domains in the polymer matrix. Higher proportions of LC (7:3) resulted in excessively saturated PDLC leading to spreading of the LC during deposition of the top electrode. This value is common in PDLC production using traditional methods^43,44^.

**Figure 3:**
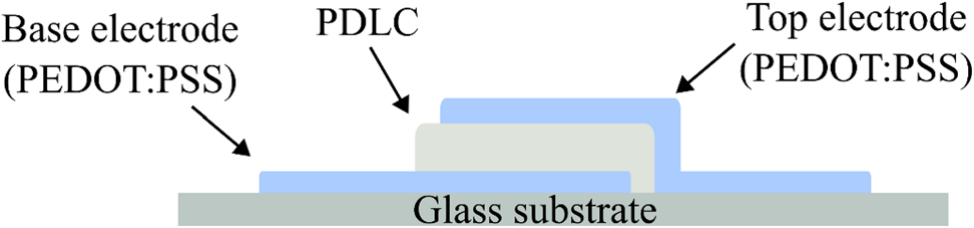
Cross-sectional representation of printed device structure. Relative dimensions are not to scale.

A cross-sectional representation of the printed device structure is shown in Fig. 3. A glass microscope slide was prepared by rinsing with IPA and acetone which acted as the substrate for the subsequent deposition. A thin film of PEDOT: PSS (Sigma 739,332), diluted 10% by weight with ethylene glycol (99.8% Merck 324,558) prior to deposition, was printed to form the base electrode and contact pad. The system was then cleaned prior to the loading of the PDLC solution to prevent contamination. A 1.4 × 1.4 mm square of the PDLC solution was deposited over the base electrode. A 200 µm overlap was used to reduce the probability of short-circuiting the base and top electrodes. The system was cleaned and the PEDOT: PSS solution was reloaded into the atomiser. The top electrode was then printed directly onto the PDLC and the device was left to dry in ambient conditions overnight.

Table 1 shows the AJP parameters used for both materials that made up the device. Direct printing onto the PDLC was achieved by the use of a nozzle with a 300 µm orifice which gave an output pressure of less than 0.15 psi. Deposition with narrower nozzles produced finer spatial resolution but caused the LC in the PDLC to spread across the substrate due to the higher output pressure (~ 1.2 psi). The 300 µm nozzle was used for all layers. Demonstration of the printed PDLC did not require the spatial resolution afforded by the narrower nozzles available. The ultrasonic atomiser was operated at full power (48 V 0.7 A) for both materials.

**Table 1:** Printing parameters for each material.

Layer material	Platen speed (mms^−1^)	Carrier gas flowrate (sccm)	Sheath gas flowrate (sccm)	Material volume in atomiser vial (ml)
PEDOT: PSS	3	25	60	1
PDLC	5	30	120	0.75

**Figure 4:**
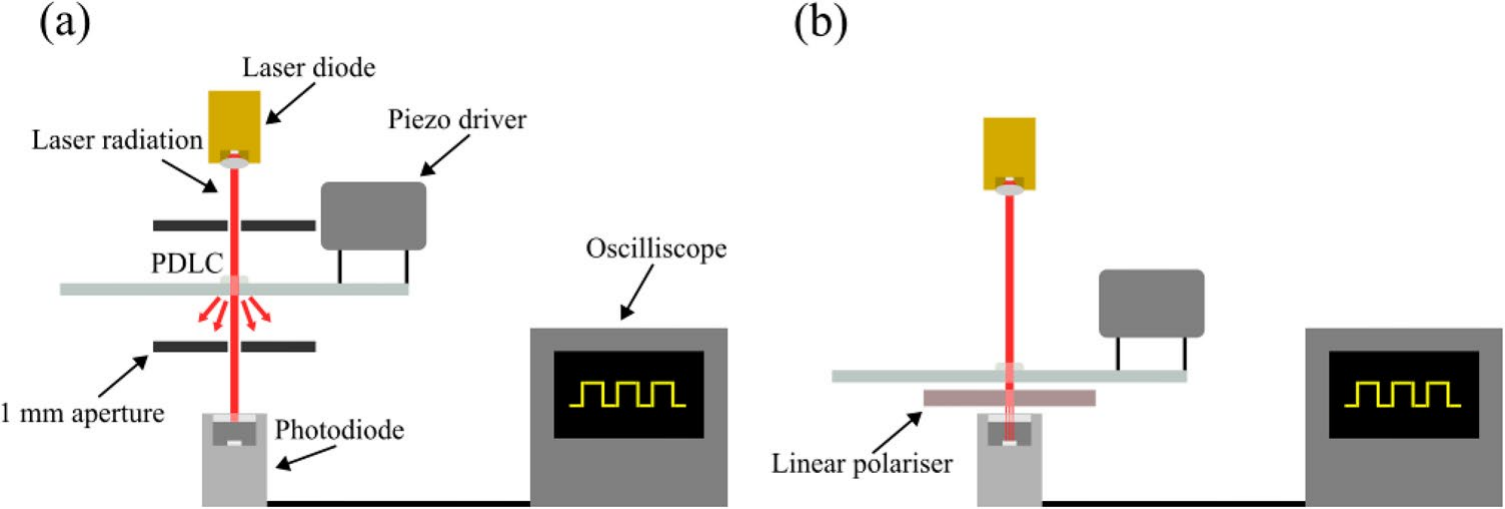
Experimental setup for: (**a**) transparency measurements; (**b**) cross-polarised transmission and relaxation time measurements.

The finished device was tested for its transparency, cross-polarised transmission, scattering and relaxation time. Radiation from a 633 nm laser diode was directed through two apertures, each 1 mm in diameter, either side of the PDLC device. Light scattered by the PDLC was baffled by the second aperture and so only specularly transmitted light was measured. An initial measurement was made with a plane glass slide identical to the substrate of the PDLC as a reference. Percentage transparency measurements were then made with reference to this value. This also accounted for the fact that the field of view of the measurement device was greater than the size of the aperture. The voltage supplied was increased and a Hamamatsu S12092-02 silicon avalanche photodiode (APD) amplifier circuit was used to measure the light that passed through the second aperture. This circuit, as described by Hobbs et.al.^45^ utilised a conventional transimpedance amplifier (TIA) configuration with a response time set to less than 3 µs. A 100 µm aperture was placed directly above the APD-TIA to reduce signal from reflections. The setup for the transparency measurements is shown in Fig. 4a.

Cross-polarised transmission was measured by removing the apertures and placing a linear polarising film 90° rotated to the plane of the laser polarisation. This was to ensure that the only laser light that was transmitted was that which was randomly re-polarised by the LC domains inside the PDLC. The APD-TIA was placed beneath the polariser such that all transmitted light was measured, and the system was isolated from exterior sources of light. A square wave voltage was supplied to the PDLC from a piezo driver board (Texas Instruments DRV2700EVM) by spring loaded pins in contact with the two printed electrodes. The piezo driver was used to output 5 discrete voltages (Vpp): 46 V, 74 V, 90 V, 116 V and 140 V. Each voltage was applied across the device for a range of frequencies (100–3200 Hz) and the output from the APD-TIA circuit was recorded. Figure 4a shows the experimental setup used for this measurement.

The relaxation time of the LC was measured using a similar experimental arrangement shown in Fig. 4b. Each voltage was pulsed with a 500 ms period and the signal from the photodiode was measured using an oscilloscope (Agilent Technologies DSO-X 2002A). The oscilloscope was used to measure the rise time of the signal, which was equivalent to the relaxation time of the LC. Each voltage was tested for the same range of frequencies as the transparency measurements.

The same device structure was printed onto multiple, non-planar surfaces (a curved lens surface and the vertex of a prism) in order to demonstrate the utility of the AJ process for non-planar LC devices. Both non-planar substrates were placed on the print stage without any special mounting required and the toolpath was executed without modification from the toolpath for printing onto the flat substrates.

Micrographs were taken of the PDLC on the prism using an FEI Nova Nano SEM in low-voltage mode using a primary beam accelerating voltage of 800 V. The prism substrate was coated with silver paint in order to establish a grounding contact during the SEM imaging. Detection of the signal was made with the through lens detector (TLD) with a suction voltage, the voltage used to attract secondary electrons for detection, of 250 V and the working distance of 4 mm.

## Results and discussion

**Figure 5:**
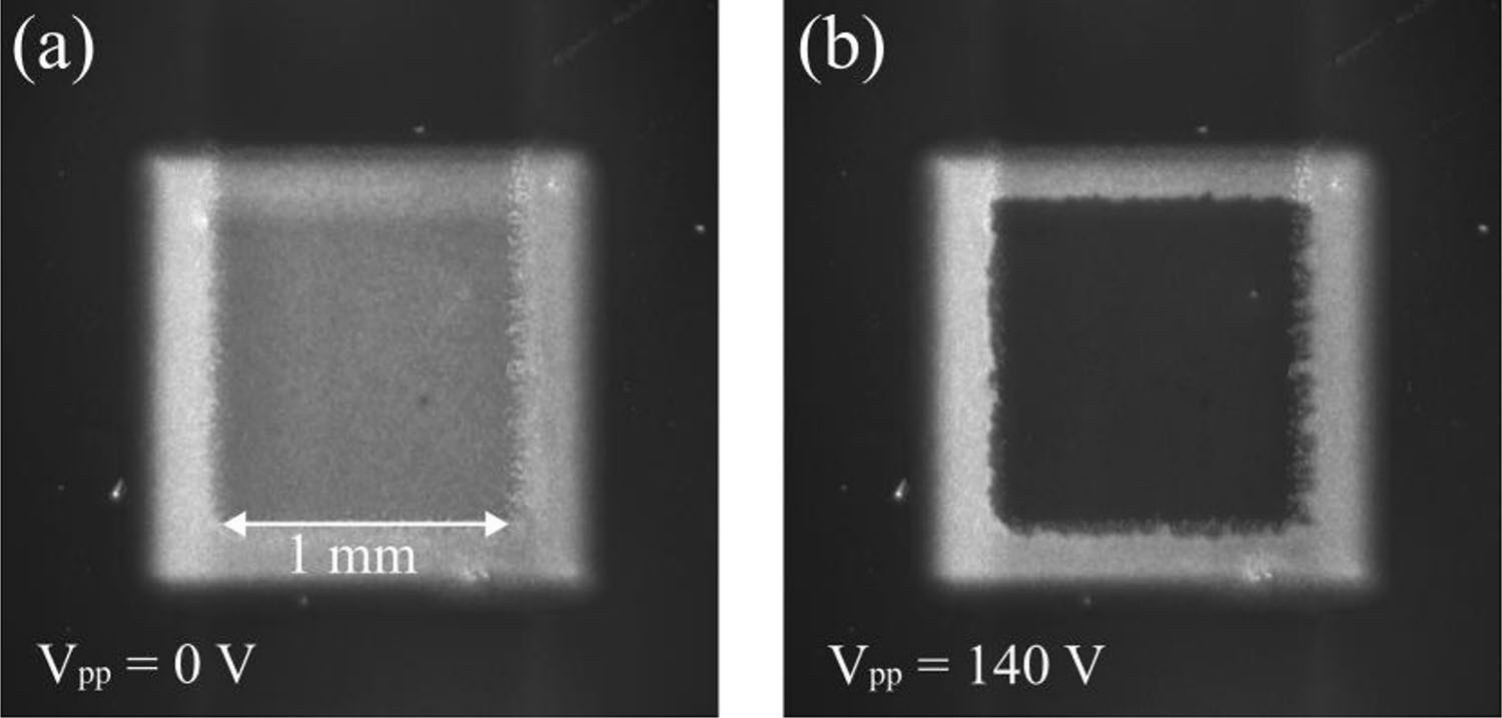
Cross-polarised microscope images of the PDLC: (**a**) with no applied AC voltage; (**b**) with and applied AC voltage of 140 V.

Figure 5 shows cross-polarised microscope images of the PDLC with no applied AC voltage and with and applied voltage of 140 V. Only the area of overlap between the two printed electrodes changes in transmission when voltage is applied. The perimeter of the active area of the device is coarse, likely due to a surface energy mismatch between the top electrode and the PDLC during deposition. This is a common problem in AJP^46^, though we believe optimisation of the PEDOT:PSS solvent compatibility with the PDLC surface energy could improve the edge quality of the top surface electrodes. Different polymers have different surface energies. It is possible there are polymers with better wetting properties that would enable more coherent films. The image shows that the PDLC diffuses light uniformly and there is no visible evidence that the top layer deposition has significantly influenced the distribution of the LC domains in host polymer.

**Figure 6:**
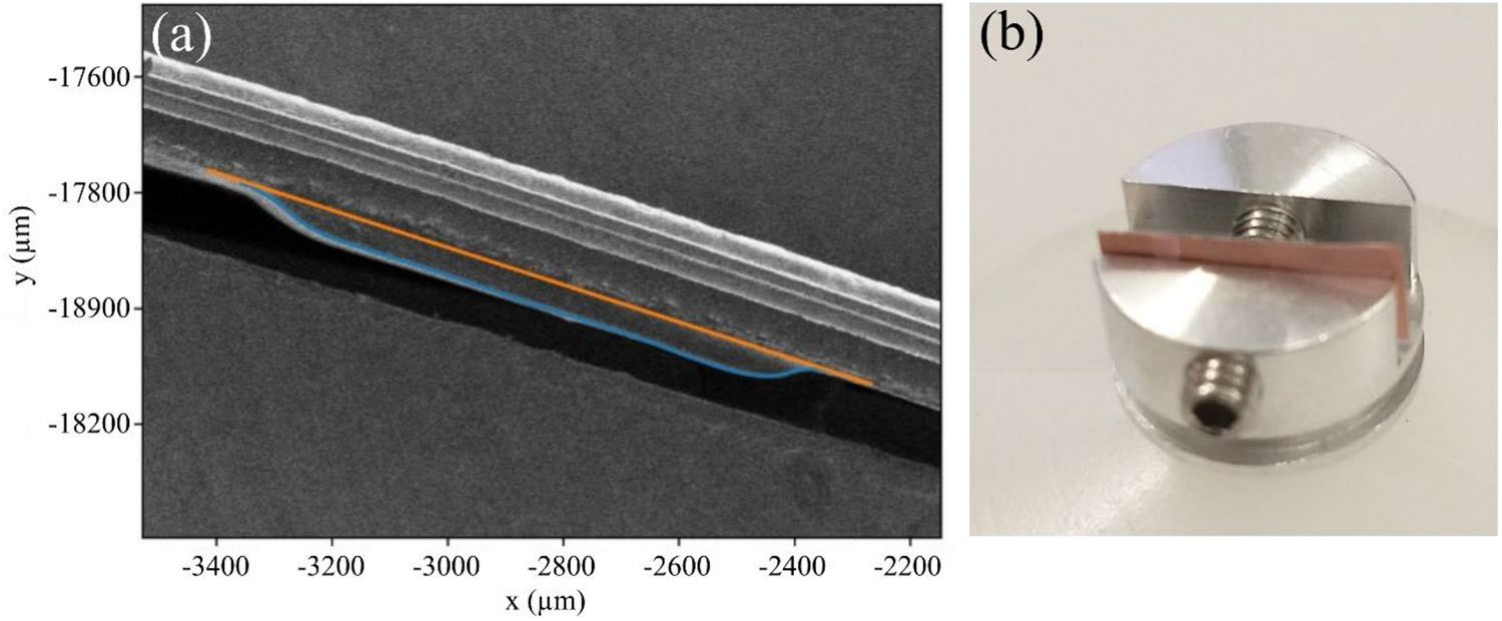
**a** shows a cross-sectional SEM image of a PDLC film on copper. The traces of the PDLC cross-section outline were made by grey-level thresholding the PDLC surface (blue line) and plotting a straight line along the copper backing interface (orange line). (**b**) shows the test sample in a cross-section holder for the SEM imaging.

Figure 6a shows an SEM image used to measure the thickness of the PDLC. The PDLC was printed on copper substrate and was cross-sectioned using a knife blade and mounted in a cross-section holder shown in Fig. 6b. A Helios NanoLab 660/G scanning electron microscope (SEM) was used to take secondary electron images of the cross-section region. The beam accelerating voltage was 1 kV and beam current 13 pA. A trace of the cross-section outline was made by grey-level thresholding (curved surface) and plotting a straight line along the copper backing interface. The average thickness of the PDLC is from 7 measurements between the copper backing (orange line) to the PDLC surface (blue line), perpendicular to the copper backing interface. The average thickness was measured to be 46.8 µm.

**Figure 7:**
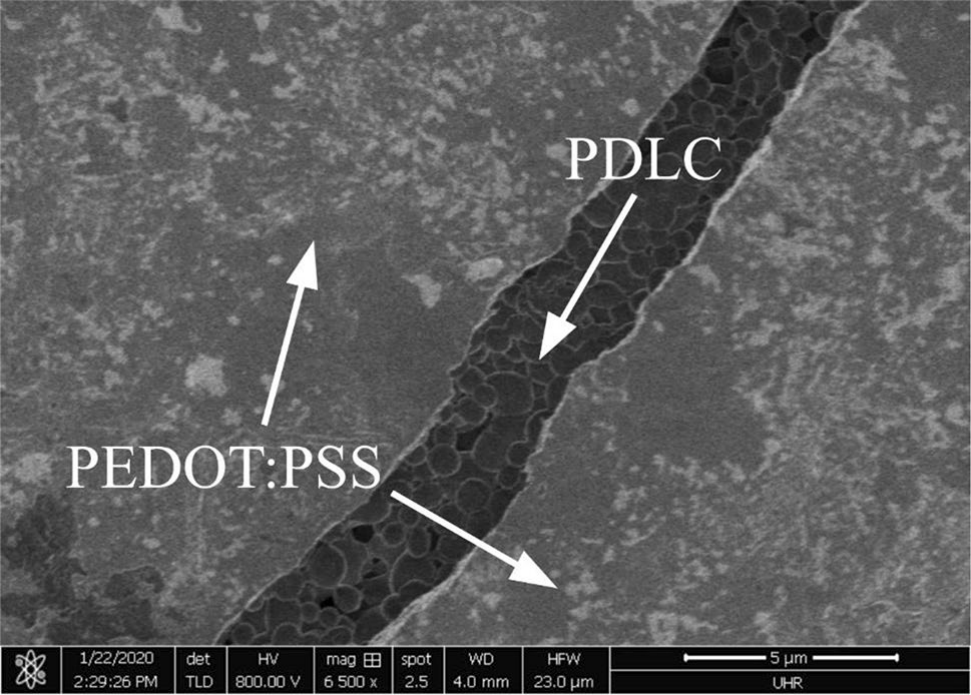
An SEM image of a crack in the top PEDOT: PSS electrode. The LC domains are visible beneath.

Although the top surface of the PDLC is coated in PEDOT:PSS, microscopic cracks that form as the polymer dries allow the PDLC to be imaged beneath using scanning electron microscopy (SEM) techniques. The image in Fig. 7 shows an SEM image of a crack in the top PEDOT: PSS electrode. The LC domains are visible beneath. Figure 7 shows one such crack on the top surface of the PDLC printed onto the prism.

The imaging reveals a series of such cracks and gaps that form during the drying. We believe this could have contributed to the high scattering of the device in the on-state and a reduced conductivity of the electrodes, reducing the electric field across the PDLC. We believe this cracking could be significantly reduced by optimising the top layer deposition and with the addition of some post-processing steps to increase the PEDOT:PSS conductivity^47,48^. We believe a lower vapour pressure solvent for the PEDOT: PSS would reduce the cracking. Slower evaporation would allow the films to form more gradually and reduce stresses within the film.

**Figure 8:**
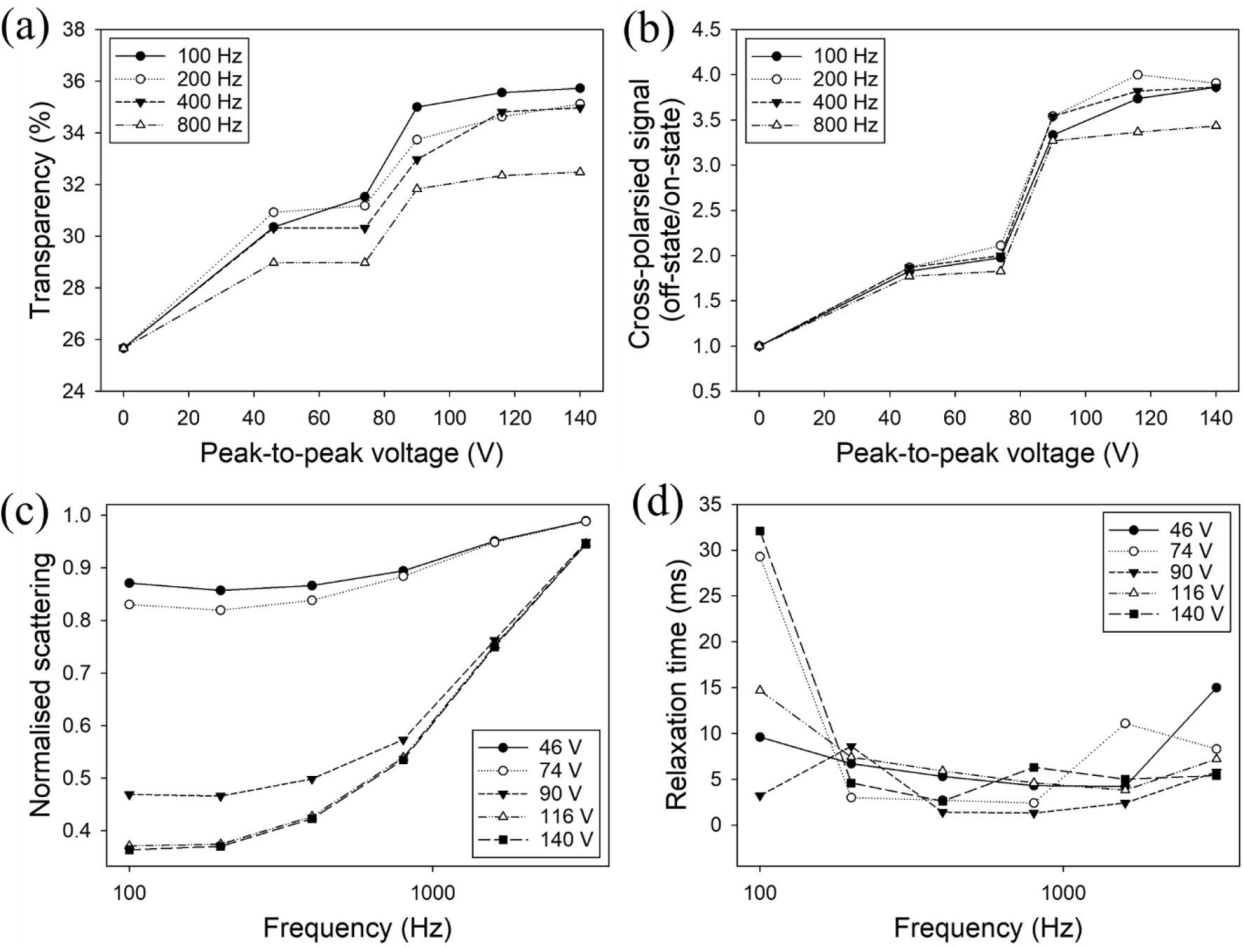
(**a**) Transparency against applied voltage for four different frequencies. (**b**) Repolarised signal normalised to off-state repolarised signal against applied voltage for four different frequencies of voltage. (**c**) Scattered light detected at an angle of 50° normalised to the off-state scattering against frequency of the applied voltage. (**d**) PDLC relaxation time against frequency for five voltages.

Figure 8a shows a graph of transparency as a function of voltage. The threshold voltage (V10) and the saturation voltage (V90) are defined as the voltage required to switch the PDLC to 10% and 90% of its maximum transparency respectively. The off-state transparency was relatively high (26%) compared to literature values (1%)^49^ with V10 less than 46 V. We believe the relatively low on-state transparency resulted from an increase in the resistance of the PEDOT: PSS electrodes due to the higher resistance of the electrodes caused by the cracking seen in Fig. 7.

The on-state transparency was measured to be 36% with a voltage of 140 V at 100 Hz. V90 for 100 Hz was between 74 and 90 V. Higher voltages were tested but are not reported here because the device performance became unstable. We believe this was caused by physical deformation of the top PEDOT: PSS layer with the application of stronger electric fields. This, and the on-state transparency, could be increased by variation of the printing parameters and requires further optimisation which was reserved for future work.

The graph in Fig. 8b shows attenuation of cross-polarised signal against voltage of the applied AC field. The PDLC was maximally transmissive due to the random polarisation of the laser radiation when there was no applied AC voltage. There is no significant decrease in random polarisation above 90 V which suggests the majority of the LC molecules are in alignment with the electric field at this voltage.

The close similarity between the relationships in Fig. 8a,b demonstrates how the LC molecular orientation affects the transparency. It can be inferred from this closeness that the low on-state transparency was not a result of poor LC alignment but possibly due to scattering caused by the top electrode. Further optimisation of the printing process could improve this.

Figure 8c shows how scattering at 50° to the axis of transmission varied with frequency for five voltages. The scattering values are normalised to the off-state signal level. The device was maximally scattering below 800 Hz, as can be seen from the graph. As frequency increases, on-state scattering approaches the off-state value for all voltages. V10 and V90 can also be inferred from the graph and are indicated by the large gap between the plots for 74 V and 90 V.

The frequency dependence of the relaxation time for the PDLC is shown in Fig. 8d. The points are the median values of nine periods to reduce the impact of outliers which occur due to the presence of the AC frequency on the APD signal waveform.

The longest relaxation times were measured at 100 Hz for most voltages and dropped off sharply as the frequency was increased to 200 Hz. The shortest relaxation times were measured between 400–800 Hz for most of the applied AC voltages and are lowest for 90 V at 1.3 ms. There is an indication of an upward trend beyond 3200 Hz.

**Figure 9:**
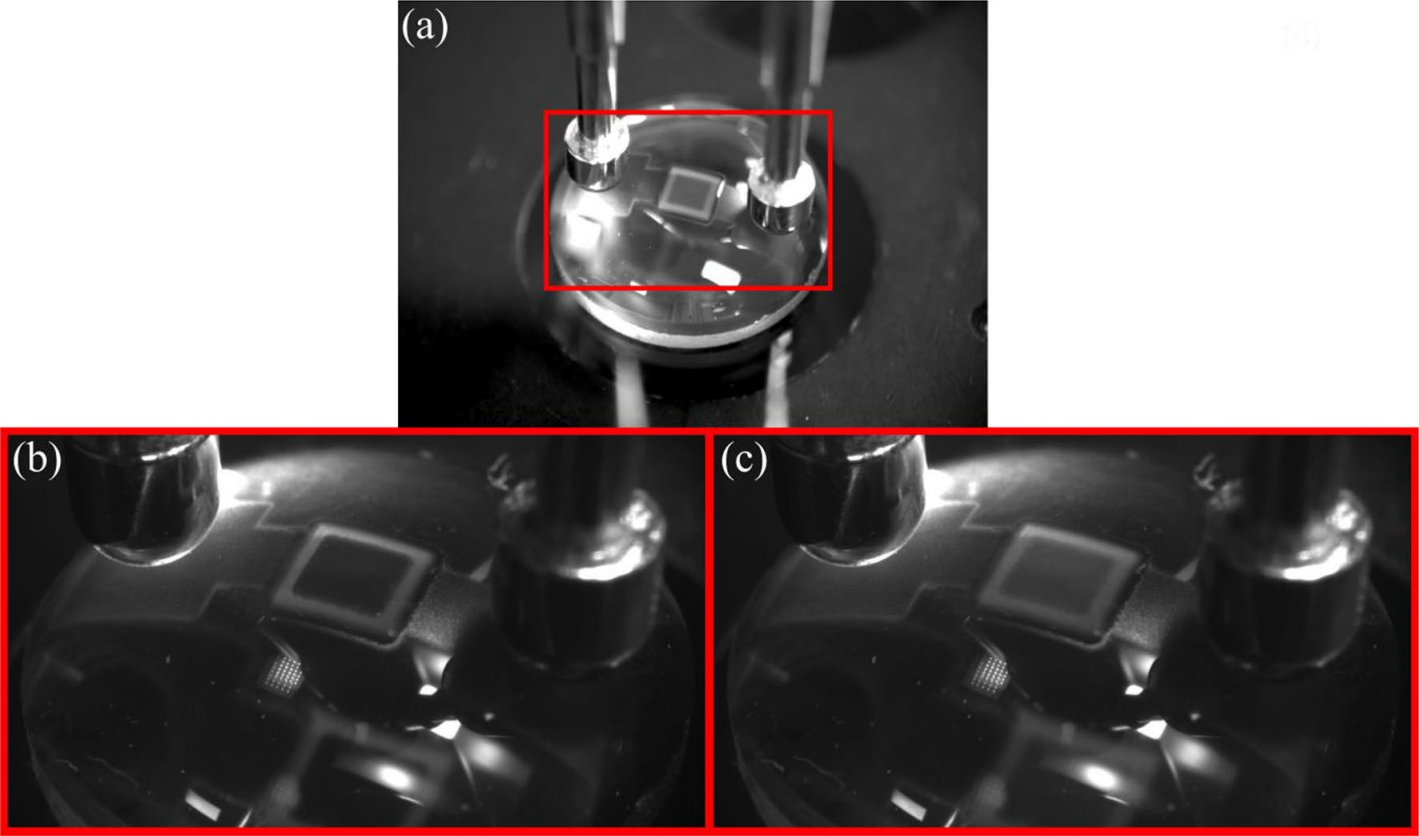
(**a**) Device structure printed onto a 9.5 mm diameter 10 mm focal length lens; (**b**) in the powered, transparent state; (**c**) in the scattering state.

One of the advantages of AJP is the ability to print on non-planar surfaces. We have demonstrated this in Fig. 9a by printing a PDLC directly onto a 9.5 mm diameter glass lens. By printing arrays of these ‘pixels’ we have the potential to control transmission through focusing optics both spatially and in optical density. This could contribute, for example, to the field of Fourier optics by shaping wavefronts away from the imaging planes.

**Figure 10:**
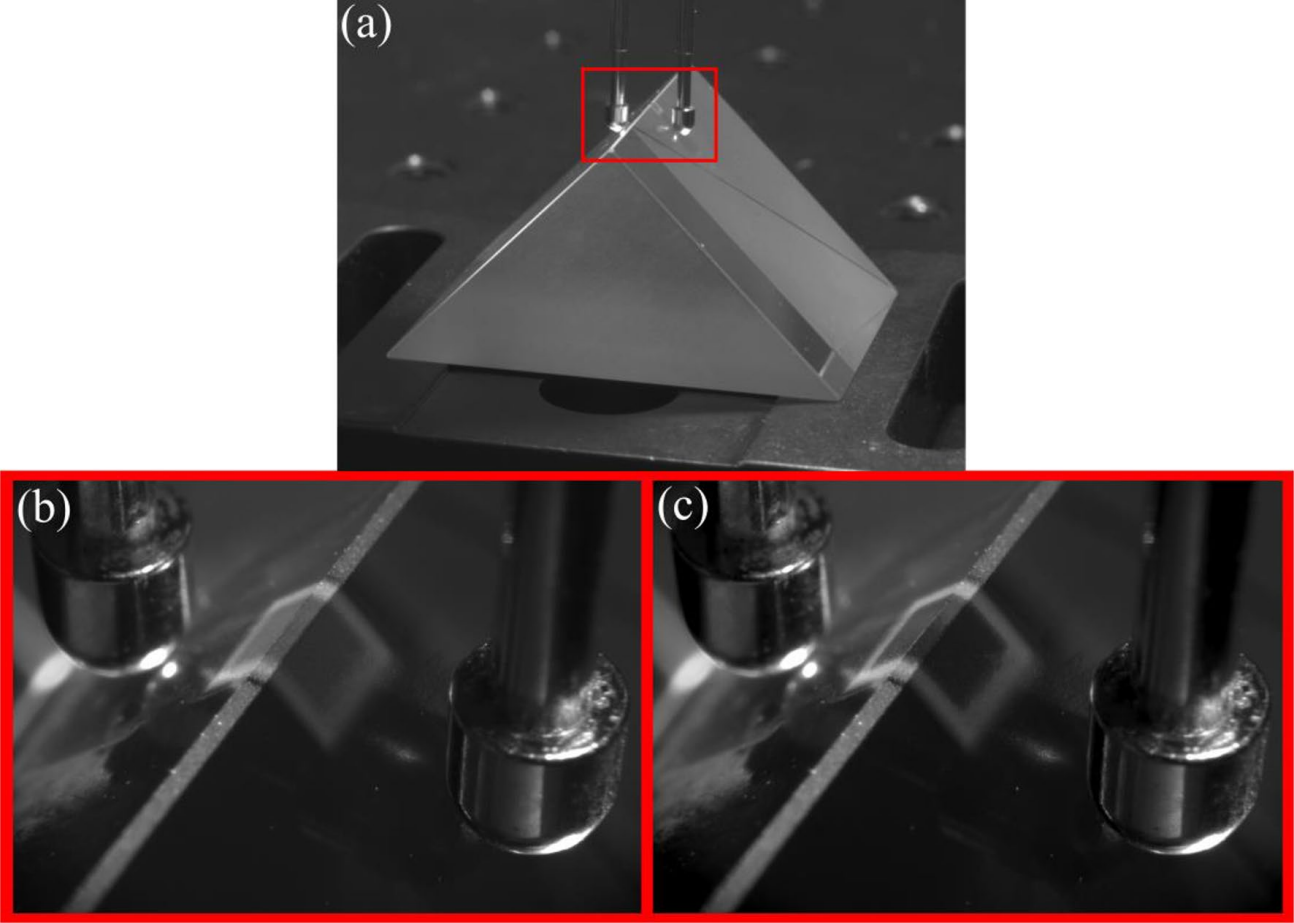
(**a**) A wide view of the prism with the printed device shown for context. The red rectangle represents the area shown in (**b**) and (**c**); (**b**) shows the device in the off-state; (**c**) shows the device in the on-state.

Figure 10 shows a demonstration of the freedom in substrate geometry selection afforded by the AJP process. We have demonstrated the flexibility of substrate geometry by printing a PDLC device directly across the 90° vertex of a glass prism. This preliminary exhibition of devices on extreme geometries paves the way to a new dimension for optical and photonic devices.

The devices printed in this work were not tested for their functionality under flexion because it was our aim to demonstrate the devices could be implemented onto nonlinear geometries in-situ. The materials used for this work are inherently compatible with flexible devices but demonstrating this is beyond the scope of this work^25,50^. Furthermore, the devices would benefit from an additional coating for protection. Physical contact would likely damage the devices due to the small thicknesses of the layers though this was not characterised in this work.

## Conclusion

Single substrate PDLCs have been demonstrated by AJP with a transparency contrast ratio of 1.6 and a relaxation time of 1.3 ms. The production method has been capitalised upon to achieve a novel device structure which precludes the need for spacers or a second substrate for an electrode. The new level of utility of production by our methods allows new PDLC device applications. It could also afford implementation of PDLCs into isolated optical systems by directly printing onto optical components. Our demonstration of printing a PDLC ‘pixel’ directly onto the curved surface of a glass lens lays the groundwork for a wide range development of AJP of LC. The deposition onto the extreme geometry of the prism vertex also allows us to hypothesise how non-planar devices could be implemented in the field of Fourier Optics. Our technique significantly expands the potential application space for PDLC devices and provides a framework for further development in the future.

## Data Availability

All relevant data are shown in the paper or could be recreated by following the methodology in the paper.
